# Pathogenesis insights from an ancient and ubiquitous spirochete

**DOI:** 10.1371/journal.ppat.1009836

**Published:** 2021-10-21

**Authors:** Jenifer Coburn, Mathieu Picardeau, Christopher W. Woods, Timothy Veldman, David A. Haake

**Affiliations:** 1 Medical College of Wisconsin, Milwaukee, Wisconsin, United States of America; 2 Pasteur Institute, Paris, France; 3 Duke University Medical Center, Durham, North Carolina, United States of America; 4 Durham VA Medical Center, Durham, North Carolina, United States of America; 5 VA Greater Los Angeles Healthcare System, Los Angeles, California, United States of America; 6 The David Geffen School of Medicine at the University of California, Los Angeles, California, United States of America; Nanyang Technological University, SINGAPORE

## Introduction

**Fig 1:**
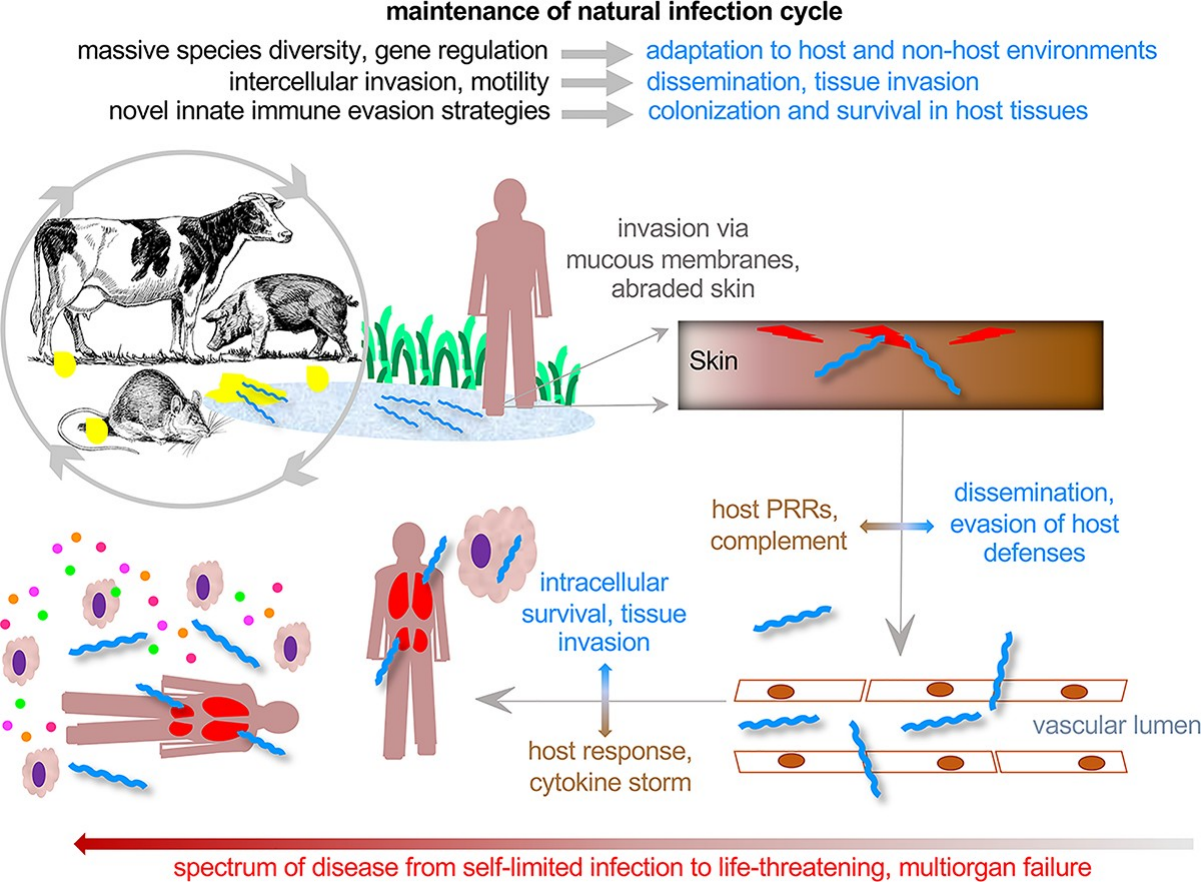
Overview of leptospirosis. Potentially human pathogenic leptospires are maintained in zoonotic infection cycles in wildlife and domestic animals. Leptospires colonize the renal proximal tubule of reservoir hosts and are shed in the urine. Urine contamination of water and mud are common sources of human exposure. In humans and disease susceptible animals, leptospires disseminate and cause symptomatic disease ranging from mild to severe and, in some cases, death.

Leptospirosis is a ubiquitous zoonotic infection caused by bacterial spirochetes that are equally adapted to life in the aqueous environment as they are to infection of their eucaryotic hosts. Leptospires owe their ubiquity to having evolved from free-living saprophytes to become nonpathogenic commensals of a wide range of mammals and, although not as well documented, birds, amphibians, and reptiles [1,2]. They colonize the proximal renal tubules of the host, in which they proliferate in the nutrient-rich glomerular filtrate, and from which they are shed into the environment by host urination. Most infections are mild or asymptomatic, but others result in organ failure and death (Fig 1). Significant impacts on human well-being have been documented, with an estimated 1 million cases and approximately 59,000 deaths per year, many of which occur in tropical, medically underserved regions of the world [3]. Leptospirosis affects not only human health but also livestock farming, causing great economic or subsistence resources losses. Despite the fact that leptospirosis has been much less investigated than other illnesses with comparable or even lower burden [4], a number of remarkable discoveries have recently emerged about these organisms and the infections they cause.

### Massive species diversity

**Fig 2:**
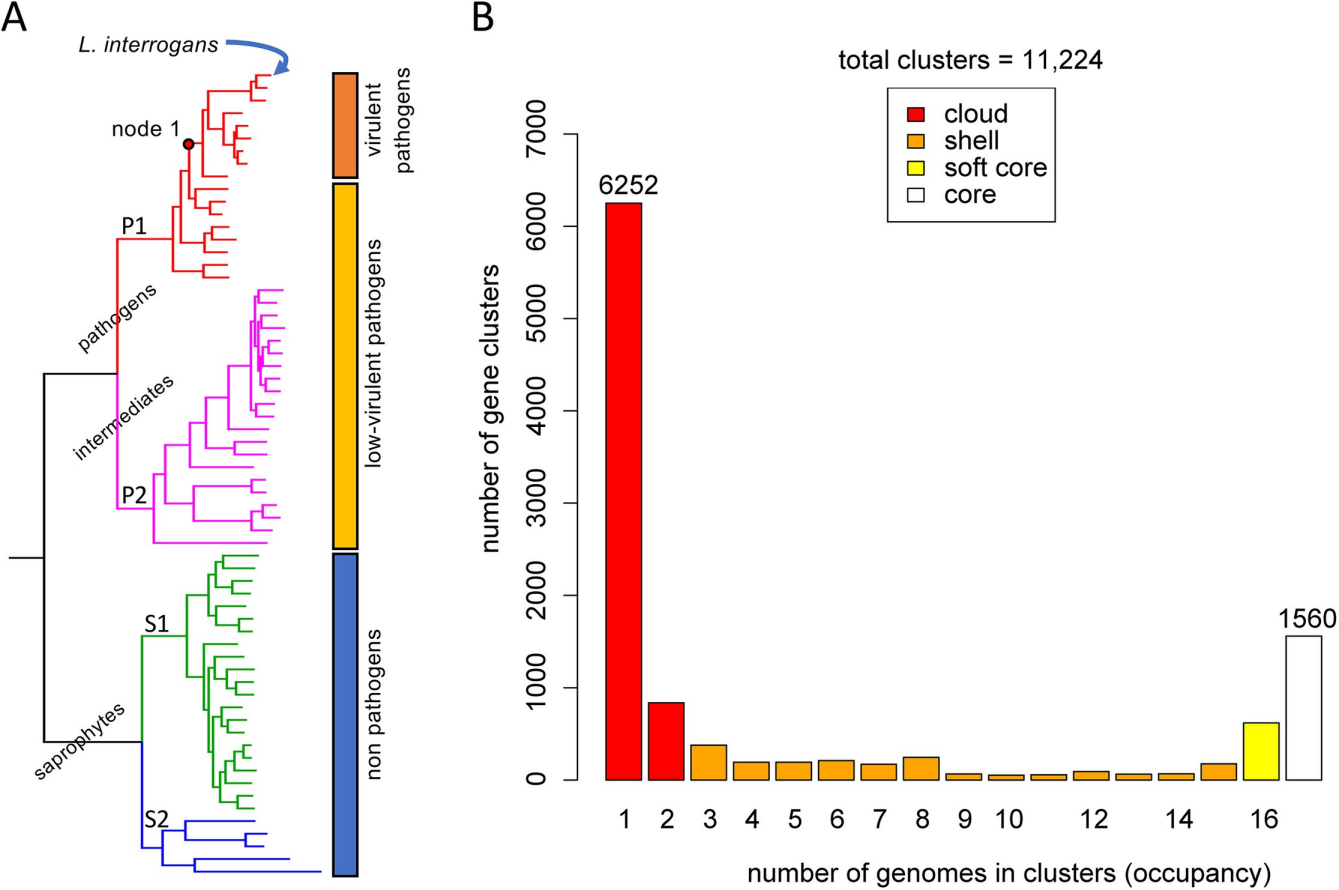
Massive species diversity. (A) Phylogenetic tree showing the relatedness of the 64 *Leptospira* species. *Leptospira* species are clustered as non-pathogens, low-virulent pathogens, and virulent pathogens according to their virulence status in animal models, prevalence in severe infections, and presence of virulence factors. Node 1 indicates the node from which descend pathogenic species are most frequently involved in human disease. (B) Distribution of gene clusters in the P1 clade revealing an open pan-genome with a relatively high number of gene clusters found only in a single species. Adapted from Vincent and colleagues [5].

With the description of many new leptospiral genomes, a striking pattern of massive species diversity has emerged: *Leptospira* species belonging to the P1 clade, which includes human pathogens, have an open pan-genome with a relatively high number of genes found only in a single species (Fig 2) [5]. The open pan-genome reflects the leptospiral life cycle, which includes the ability of leptospiral pathogens to form biofilms to withstand environmental stress and survive for prolonged periods in milieux such as soil and aqueous habitats [6]. Such settings contain complex microbial and chemical compositions, facilitating a high rate of horizontal gene transfer that enables reworking of cellular functions to allow rapid adaptation to new environmental conditions and hosts. Leptospiral genes and gene fragments are derived both from unrelated bacteria and other leptospires, resulting in mosaic genes with diverse phylogenetic ancestry [7,8]. Clues to which pathogen-specific genes are required for virulence have been obtained from RNAseq studies examining differential expression during adaptation to the mammalian host [9]. Determining the impact of specific genes on in vivo fitness has also been determined by high-throughput sequencing of tissues from animals challenged with transposon mutant pools [10]. Targeted mutagenesis in leptospiral pathogens has been challenging and has only recently become more reliable through the development of the CRISPR dCas9 and transcription activator-like effectors (TALEs) as gene silencing approaches [11,12]. These novel tools made it possible to show that the pathogen-specific, multifunctional **l**eptospiral **i**mmuno**g**lobulin-like domain (Lig) proteins were required for virulence [11,12].

### Rapid dissemination

Rapid and widespread dissemination to all organs, including the eye and brain, is a hallmark of leptospirosis. Remarkably, *Leptospira interrogans* is detectable in all organs of perfused animals within 1 hour after intraperitoneal inoculation of hamsters [13]. Consistently, flagellar mutants with decreased motility are attenuated for virulence in animal models [14,15]. Leptospires are uniquely equipped for dissemination through their corkscrew morphology, 200 nM diameter (in Greek, leptos means “thin”), and powerful propulsion by their endoflagella. As in other spirochetes, the organs of motility are called “endoflagella” because they are not surface exposed; instead, they are entirely within the periplasm. Leptospires are unique in that they have a single supercoiled endoflagellum extending axially toward the middle of the cell from a flagellar motor embedded in the inner membrane at each pole, without overlap. Endoflagellar rotation imparts a swimming motility that enables leptospires to be particularly invasive at liquid–gel borders, such as the interface between the vascular lumen and the endothelium, with 30 times the swimming force of *Escherichia coli* [16]. Recent elegant high-resolution cryo-electronic tomography studies have shown that the function of leptospiral endoflagellar filaments relies on an asymmetric sheath that imparts their supercoiled structure [17].

### Host tissue barrier invasion

Another key to efficient transmigration across host tissue barriers appears to be targeting of E- and VE-cadherins, which are integral to the intercellular adherens junctions of epithelium and endothelium, respectively [18,19]. Virulent *L*. *interrogans*, but not the saprophytic *Leptospira biflexa*, disrupt adherens junctions of endothelial and epithelial cells in vitro, resulting in loss of VE- and E-cadherins and disruption of the associated catenins, which link cadherins to the intracellular actin cytoskeleton. A clue to the molecular mechanisms for endothelial barrier disruption emerged from a study examining the crystal structures of 4 *L*. *interrogans* leucine-rich repeat (LRR) proteins [20]. As has been observed with other LRR proteins, the 23-residue repeating leucine-rich motifs formed a characteristic curved solenoid structure. One of these LRR proteins, LIC10831, has a structure and binding pocket similar to that of the *Listeria monocytogenes* internalin InlA. InlA mediates the first step of listerial invasion of the intestinal epithelium by binding to unpaired E-cadherin proteins as they become accessible through the normal sloughing of intestinal epithelial cells at the tip of the brush border. LIC10831 is actively secreted by *L*. *interrogans* and specifically binds E- and VE-cadherins at a binding coefficient 10-fold lower than InlA [21]. Importantly, LIC10831, but not an LRR protein (LIC12234) that bound other host factors, bound specifically to the cell–cell junctions of endothelial cells. Two other VE-cadherin adhesins were identified using phage display [22], suggesting that targeting of endothelial barrier integrity may be a key feature of pathogenic *Leptospira* species.

### Evasion of innate immune recognition

Reflecting their ancient history of coevolution with eukaryotes, pathogenic leptospires have evolved an array of novel strategies to evade and/or alter the innate immune response. These strategies may well be important in extending the time of persistence in the renal tubules of reservoir hosts for shedding in the urine. Leptospires are stealth pathogens that evade recognition with altered microbial-associated molecular patterns (MAMPs) structures [23]. For example, *Leptospira* spp. escape murine TLR5 recognition through the peculiar subsurface localization and stability of the FlaB flagellar subunits, combined with specific down-regulation of FlaB transcription during mammalian infection [23]. An additional immune evasion strategy involves leptospiral lipoproteins that bind MAMPs and block their recognition by host pattern recognition receptors (PRRs) of the Toll- and NOD-like families. In some cases, these PRR-blocking lipoproteins are some of the most abundant proteins in pathogenic leptospires. This strategy applies to leptospiral lipid A, which is similar to gram-negative endotoxin but differs structurally in key ways such that, although it is recognized by mouse TLR4, it is not recognized by human TLR4 [24]. In fact, C3H/HeJ mice lacking TLR4 are more susceptible to leptospiral infection have a higher leptospiral burden than C3H mice with an intact TLR4 [25]. However, even in mice with intact TLR4, leptospiral lipopolysaccharide (LPS) O-antigen and multiple LPS-binding lipoproteins reduce TLR4-mediated uptake by macrophages and their TRIF-dependent activation [26]. Similarly, LipL21, a major leptospiral lipoprotein has now been recognized as a peptidoglycan-binding lipoprotein that enables escape from NOD1 and NOD2 recognition [27].

### Evasion of innate killing mechanisms

Leptospires have evolved a variety of strategies for evasion of host innate killing mechanisms. These include escape from complement by surface presentation of LigA, LigB, and other proteins that bind host complement regulators [28,29]. Pathogenic leptospires also express catalase, encoded by *katE*, which is required for resistance to reactive oxygen species (ROS) and for virulence in the hamster model [30]. Expression of *katE* is transcriptionally controlled by the peroxide stress regulator PerR, a novel H_2_O_2_ sensor [31–33]. Recently, a second potential PerR was identified, called PerRB, which is present only in pathogenic *Leptospira* species. Inactivating *perRA* or *perRB* led to an increased tolerance to 2 different components of the phagocytic oxidative burst, H_2_O_2_ and superoxide, respectively, indicating that the 2 regulators do not have a redundant functions [34]. While single *perRA* and *perRB* mutants were virulent for hamsters, the double mutant was avirulent for hamsters [34]. These results indicate that, although the double mutant has the metabolic pathways required for infection, it lacks expression of specific virulence-related gene products. Interestingly, RNAseq and protein expression studies involving *perRA* and *perRB* single and double mutants revealed complex regulons, including many coding and noncoding RNAs, some of which are likely unrelated to resistance to oxidative stress [34].

### Rapid diagnostics

Leptospirosis is a common cause of acute febrile illness in areas where dengue, malaria, and rickettsial infections are also common. Though the majority of cases are mild, life-threatening leptospirosis is common in many tropical countries with a case fatality rate exceeding that of other common febrile illnesses. Our ability to identify the etiology of febrile illness and provide targeted therapy is limited by the nonspecific clinical presentations, low sensitivity of molecular methods, and low specificity and delayed utility of serological methods. Standard biomarkers such as C-reactive protein (CRP) and procalcitonin have relatively low specificity for leptospirosis [35]. In contrast, the transcriptional response to leptospirosis and scrub typhus is distinguishable from that of patients with viral etiologies [35]. Likewise, proteomic approaches utilizing multiple inflammatory biomarkers such as serum amyloid A and leucine-rich alpha-2 glycoprotein provide diagnostic utility greatly superior to CRP or PCT alone and are more readily translated to rapid diagnostic platforms suitable for use in low resource healthcare settings. Host response signatures and biomarkers such as decreased cathelicidin may also enable prediction of disease severity among patients with early infection [36] and identify patients who would benefit from antibiotics and other interventions.

## Conclusions

While much more work is needed to validate and expand these discoveries, leptospirosis research is providing unprecedented insights regarding these ancient pathogens and their host interactions. Further elucidation of leptospiral genetic diversity and its global health impacts are needed, both in humans and in both domestic and wild animals using a One Health perspective. The coevolution of leptospires with their eukaryotic hosts is reflected in their novel mechanisms for immune evasion and escape from innate killing strategies. A better understanding of the remarkably rapid and efficient dissemination process will provide opportunities for improved prevention strategies including vaccine development. Finally, host response and biomarker approaches to diagnosis are needed to rapidly diagnose suspected cases and expedite their management to improve patient outcomes. A better disease burden estimate of causes of fever, including leptospirosis, in developing countries would also be most useful for policy and decision makers.
